# Duplication and Remolding of tRNA Genes in the Mitochondrial Genome of *Reduvius tenebrosus* (Hemiptera: Reduviidae)

**DOI:** 10.3390/ijms17060951

**Published:** 2016-06-16

**Authors:** Pei Jiang, Hu Li, Fan Song, Yao Cai, Jianyun Wang, Jinpeng Liu, Wanzhi Cai

**Affiliations:** 1 Department of Entomology, China Agricultural University, Beijing 100193, China; jiangp2006@126.com (P.J.); fansongcau@gmail.com (F.S.); caiyao2010@126.com (Y.C.); wjy-1989@163.com (J.W.); 2 Markey Cancer Center, University of Kentucky, Lexington, KY 40536, USA; jinpeng.liu@uky.edu

**Keywords:** mitochondrial genome, tRNA gene rearrangement, TDRL model, Reduviidae

## Abstract

Most assassin bugs are predators that act as important natural enemies of insect pests. Mitochondrial (mt) genomes of these insects are double-strand circular DNAs that encode 37 genes. In the present study, we explore the duplication and rearrangement of tRNA genes in the mt genome of *Reduvius tenebrosus*, the first mt genome from the subfamily Reduviinae. The gene order rearranges from CR (control region)*-trnI-trnQ-trnM-ND2* to CR*-trnQ-trnI2-trnI1-trnM-ND2*. We identified 23 tRNA genes, including 22 tRNAs commonly found in insects and an additional *trnI* (*trnI2*), which has high sequence similarity to *trnM*. We found several pseudo genes, such as pseudo-*trnI*, pseudo-CR, and pseudo-*ND2*, in the hotspot region of gene rearrangement (between the control region and *ND2*). These features provided evidence that this novel gene order could be explained by the tandem duplication/random loss (TDRL) model. The tRNA duplication/anticodon mutation mechanism further explains the presence of *trnI2*, which is remolded from a duplicated *trnM* in the TDRL process (through an anticodon mutation of CAT to GAT). Our study also raises new questions as to whether the two events proceed simultaneously and if the remolded tRNA gene is fully functional. Significantly, the duplicated tRNA gene in the mitochondrial genome has evolved independently at least two times within assassin bugs.

## 1. Introduction

Mitochondria are not only organelles with their own genetic material that provide energy for cells, mitochondria are also involved in the control of the cell cycle and cell growth [1]. The typical mitochondrial (mt) genome of animals usually consists of a single circular chromosome with 37 genes (13 protein-coding genes, two ribosomal RNA genes, and 22 transfer RNA genes) and a control region (CR) [2]; however, gene order is usually different between major groups [3]. With the rapid development of sequencing techniques, an increasing number of complete mt genome sequences have been determined, which has made mt genomes a popular molecular marker for inferring ancient evolutionary relationships. As much, mt has become the model for studying the mechanism of genome rearrangement [4,5,6,7,8].

Most insect mt genomes have the same gene arrangement as *Drosophila yakuba* which is acknowledged to be the ancestral pattern for insects [1,9]; however, there have been reports of gene rearrangements in many taxa, such as thrips [10,11], lice [12,13,14], wasps [15,16], and unique-headed bugs [17]. Within these groups, there are reports of rearrangements involving tRNA genes, protein-coding genes (PCGs) and even fragmented mt genomes. Among the several mechanisms proposed to explain gene order rearrangements [5,6,18], the tandem duplication/random loss (TDRL) model is most often used, and is generally considered the most recognized explanation for this occurrence in insects. According to the TDRL model, random deletion of redundant duplicated genes results in novel gene orders [18,19]. Which genes are lost depends on whether the random mutations affect the normal function of genes, and whether the derived pseudo gene will be eventually deleted from the genome [3,20]. In addition to the TDRL model, mechanisms including transposition [19], tandem duplication/nonrandom loss (TDNR) [20], inversion [21], tRNA mis-priming [22], tRNA duplication/anticodon mutations [22,23], and intramolecular recombination [24] have been proposed to account for mt gene rearrangements that we cannot easily or lucidly explain by relying only on the TDRL model.

Assassin bugs (Reduviidae), the second largest family of Heteroptera (true bugs), include close to 7000 species in almost 1000 genera and 25 subfamilies [25]. Presently, the mt genomes of 12 assassin bugs from seven subfamilies have been sequenced. Eleven of these 12 mt genomes contain the typical 37 genes and are arranged in the hypothesized ancestral gene order of insects; however, the mt genome of *Brontostoma colossus* has 38 coding genes, including the typical 37 genes and an extra *trnR* [26].

In this study, we report the complete mt genome sequence of *Reduvius tenebrosus* (*R. tenebrosus*), the first representative mt genome of the subfamily Reduviinae. The mt genome of *R. tenebrosus* has novel arrangements in the genomic region between the CR and *ND2* (*Q-I2-I1-M*). The presence of pseudo genes, the positions of intergenic spacers, and a copy of tRNA genes provide evidence that this novel gene order can be explained by the TDRL model and the tRNA duplication/anticodon mutation mechanism. We also sequenced mt genome fragments between the CR and the *ND2* of two other species (*Reduvius gregorgi* and *Acanthaspis cincticrus*) to further explore whether gene rearrangement is a common feature shared among the Reduviinae.

## 2. Results and Discussion

### 2.1. The Mitochondrial (mt) Genome of the Assassin Bug, Reduvius tenebrosus

**Figure 1:**
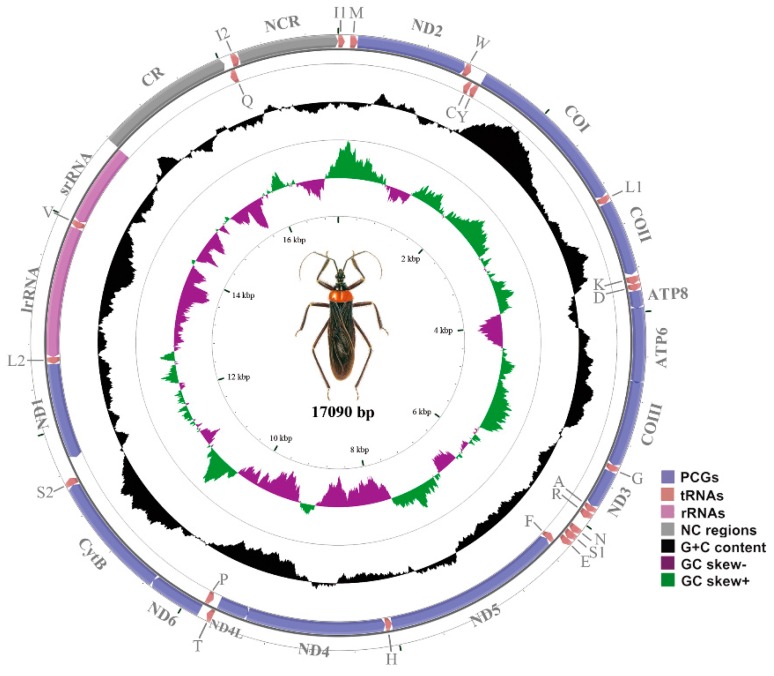
The mitochondrial genome of *Reduvius tenebrosus*. Arrows indicate the orientation of gene transcription. Abbreviations of gene names are: *ATP6* and *ATP8* for adenosine triphosphate (ATP) synthase subunits 6 and 8; *COI–III* for cytochrome oxidase subunits 1–3; *CytB* for cytochrome b; *ND1–6* and *ND4L* for nicotinamide adenine dinucleotide hydrogen (NADH) dehydrogenase subunits 1–6 and 4L; *srRNA* and *lrRNA* for large and small rRNA subunits; trnX (where X is replaced by one letter amino acid code of the corresponding amino acid), for transfer RNA (L1: CUN; L2: UUR; S1: AGN; S2: UCN; I1 and I2 indicate two *trnI* in different positions). The plotted GC content is the deviation from the average GC content of the entire sequence. The GC-skew was plotted as the deviation from the average GC-skew of the entire sequence. The inner cycle indicates the location of genes in the mitochondrial (mt) genome.

The mt genome of *R. tenebrosus* is a circular DNA molecule 17,090 bp in length. It contains 38 coding genes (13 PCGs, two rRNAs and 23 tRNAs) and a 1251-bp CR (Figure 1; Table S1). In addition to the typical 37 genes commonly found in arthropod mt genomes, an additional *trnI* (named *trnI2*) was identified in this genome. Twenty-four genes are encoded on the majority strand (J-strand) and the other 14 genes are annotated on the minority strand (N-strand). In addition to the CR, we observed 11 non-coding regions (NCR) ranging from 1 to 917 bp. We identified 11 gene overlaps, ranging from 1 to 14 bp in length (Table S1).

The nucleotide composition of the *R. tenebrosus* mt genome displays bias toward A and T (J-strand: A = 39.7%, T = 27.5%, G = 12.4%, C = 20.4%, see Table S2). In fact, the overall AT content is lower in comparison with the other 12 mt genomes of assassin bugs (Table S3). The mt genome exhibits obvious AT-skew (0.18) and GC-skew (−0.24), which is in accordance with the common strand bias of insect mtDNA [27]. Genome-wide patterns of base composition may be shaped by the highly asymmetric effects of transcription on mutagenesis, including unequal exposure of the strands to DNA damage and differential chance for repair [28]. The codon usage of PCGs also contributes to a genome-wide bias toward AT (see Table S4). At the third codon position, A or T are overwhelmingly overrepresented compared to G or C. Ultimately, the overrepresentation of A and T is attributable to nucleotide compositional bias. The six most prevalent codons are the AT-rich codons, ATT, ATA, TTA, TTT, AAT, and TAT.

**Figure 2:**
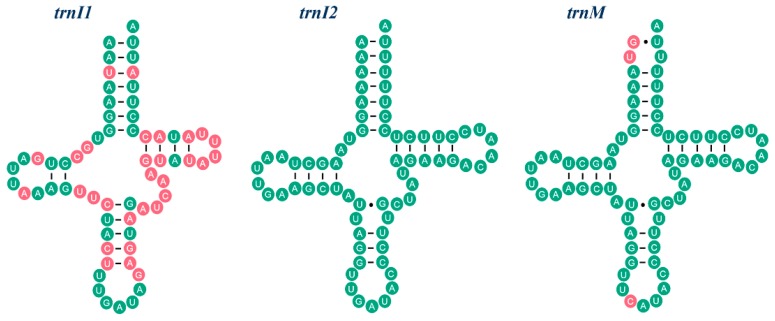
The secondary stem-loop structure and sequence similarity of *trnI1*, *trnM* and *trnI2*. Inferred Watson-Crick bonds are illustrated by lines, whereas GU bonds are illustrated by dots. Sequences of *trnI1* and *trnM* are compared with *trnI2* respectively. The identical nucleotides with *trnI2* are labeled with green circles. The variable nucleotides are highlighted in red.

The typical 22 tRNAs commonly found in insects and the additional *trnI* (*trnI2*) are present in *R. tenebrosus*. The lengths vary between 60 and 71 bp. It is worth mentioning that the sequences of *trnI2* and *trnM* only differ in three nucleotide positions, whereas the similarity between sequences of *trnI1* and *trnI2* is relatively low (Figure 2). The typical clover-leaf structure can be predicted in 22 tRNA genes, with the exception of *trnS1* (in which the dihydrouridine, DHU, arm is reduced to a simple loop, as is true in most true bugs) [17,29,30]. The locations and lengths of rRNA genes are similar to those of other assassin bug mt genomes. For instance, *lrRNA* is located between *trnL2* and *trnV* and is 1255 bp long. *srRNA* is flanked by *trnV* and the CR and is 784 bp long. The existence of the extra *trnI* raises the issue of whether both of the *trnI* function normally simultaneously. Both trn*I1* and trn*I2* may be functional genes for that both of them can form an intact secondary structure of standard mitochondrial tRNA genes [31,32] and have the identical anticodon sequence (GAU). Beyond that, the number of the ATC codon of *R. tenebrosus* tends to be higher than that of the other 12 species, and the amount of ATT codon is obviously lower compared to others. However, the total number of isoleucine codons (ATT + ATC) in *R. tenebrosus* (365) has no significant difference when compared to other sequenced assassin bugs, and the statistical test shows that the relative frequencies of ATT *vs.* ATC codons in both *R. tenebrosus* and *Triatoma dimidiata* mt PCGs strongly deviate (*p* < 0.001) from the average value of the other 13 assassin bugs (Table S3).

Moreover, the AT contents of *R. tenebrosus* and *T. dimidiate* are the lowest two of the 13 sequenced species. To determine whether this codon usage deviation is due to the duplication of isoleucine tRNAs or the relatively high GC content, we calculated the ratios of codons ending in G or C *vs.* the total codons of each amino acid in the 13 assassin bugs (Table S5). The results indicate that the preference of codons ending in G or C than A or T is evident for almost all codon boxes in *R. tenebrosus*. Therefore, the strong deviation in the relative frequency of ATT *vs.* ATC codons in *R. tenebrosus* may be related to genome-wide bias in base composition. Ultimately, the functions of the two *trnI* require more verification, such as transcriptome analysis.

Unconventional start codons in the mt PCGs of insects have been reported in previous studies, such as the start codon GTG in *ND5* [33] and *ND1* [30], TTG in *COI* [29,30] and *ND3* [34], and the tetra nucleotide start codon ATAA in *ND4* [10] and *COI* [35]. In the mt genome of *R. tenebrosus*, GTG is found as start codon in both *ND1* and *ND4L*. Other PCGs all initiate with ATN (Table S6). Furthermore, four PCGs (*COI*, *COIII*, *ND5*, *ND4*) have incomplete stop codons (a single T), which may be made into a completed and proper TAA stop codon by RNA polyadenylation during the transcript process [36]. The other PCGs terminate with either TAA or TAG (Table S6). The use of start and stop codons highlights the similar patterns in the 13 sequenced mt genomes of assassin bugs (Table S6).

### 2.2. NonCoding Regions, Pseudo Genes and a Novel Gene Order in the Reduvius tenebrosus mt Genome

**Figure 3:**
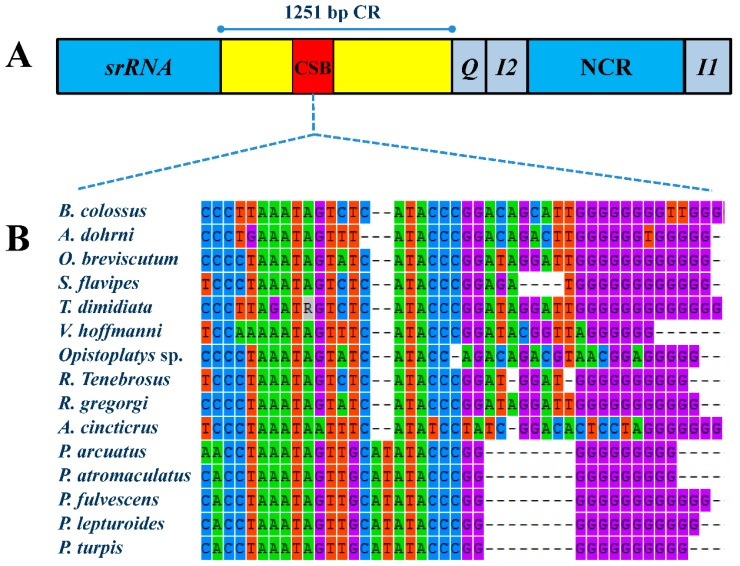
The conserved sequence block in the control region of assassin bug mt genomes: (**A**) the organization of the control region in the *R. tenebrosus* mt genome; and (**B**) the conserved sequence block in the control region of the sequenced assassin bug mt genomes. CR is the abbreviation for control region and CSB is the abbreviation for conserved sequence block. Four bases are labeled in different colors: Adenine (A)—green, Guanine (G)—purple, Thymine (T)—red and Cytosine (C)—blue.

There are a total of 2441 bp NCRs (also referred as intergenic spacers, IGS) in the mt genome of *R. tenebrosus*. The 1251 bp of the CR is the longest NCR. It is located between the *srRNA* and the *trnQ*, and has 66.2% of the AT content. We failed to detect tandem repeat sequences, which is one of the common characteristics of assassin bug control regions [33,37]. However, the sequence alignments of 15 assassin bug control regions (two of which were sequenced fragments between CR and *ND2*, see below) indicate the presence of a conserved sequence block (CSB), including a G element of 10 bp (Figure 3B), which has been reported in most assassin bugs, as well as some dipterans (referred to as G islands) [38]. They are generally thought to play a role in the replication mechanism [39].

**Figure 4:**
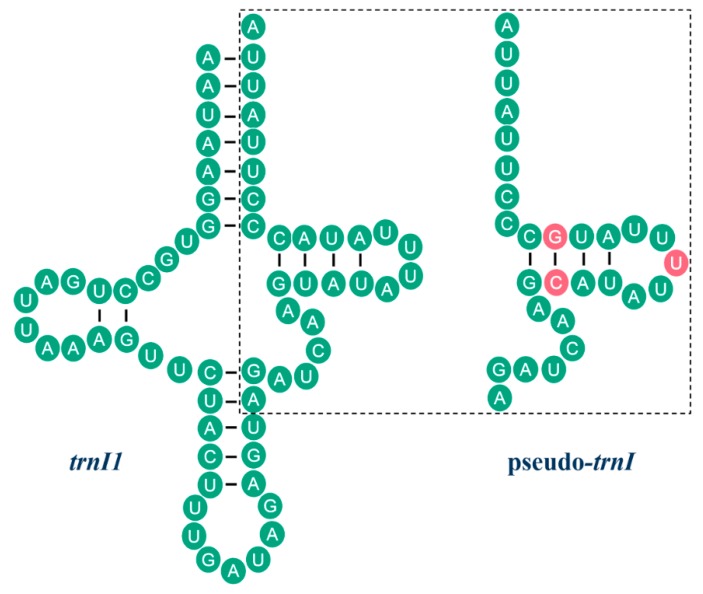
The secondary stem-loop structure and sequence similarity of *trnI1* and pseudo-*trnI*. Inferred Watson-Crick bonds are illustrated by lines, whereas GU bonds are illustrated by dots. The identical nucleotides between *trnI1* and pseudo-*trnI* are labeled with green circles. The sequences in the dotted box represent the homologous sequences between *trnI1* and pseudo-*trnI*. The variable sites are in red.

**Figure 5:**
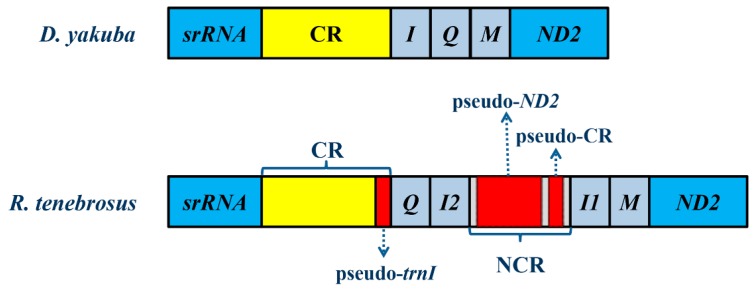
Gene rearrangement and the locations of three pseudo genes in the region between the control region and *ND2* in *R. tenebrosus*. Three pseudo genes are labeled with dotted arrows. CR is the abbreviation for control region and NCR is the abbreviation for non-coding region.

Apart from the CR, there is a relatively long NCR (917 bp) located between two *trnI* genes (Figure 3A), and 10 short NCRs ranging from 1 to 145 bp long (Table S1). In the long NCR, a 605 bp region shares significant similarity (82.26%) with the homologous sequences in *ND2*. Additionally, the 197 bp region is almost identical to its counterpart in the CR, except for five point mutations (Figure S1). We also identified a 28 bp region at the end of the CR which is similar to the sequences near the 5’ end of *trnI* (Figure 4). The significant sequence similarities between these regions indicate the presence of pseudo genes in the CR, *ND2*, and *trnI*. Therefore, we define these three regions as pseudo-CR, pseudo-*ND2*, and pseudo-*trnI*, respectively (Figure 5).

Heteroptera, also called true bugs, with the largest number of published complete mt genomes in the order Hemiptera, show only gene rearrangement events in a few taxa (Table 1), such as unique-headed bugs [29], pyrrhocoroid bugs [40] and flat bugs [41,42]. In the mt genome of *R. tenebrosus*, we found the presence of an extra *trnI* in the tRNA gene rearrangement between CR and *ND2*. This rearrangement changed the ancestral gene order of CR-*I-Q-M-ND2* to the novel gene order of CR-*Q-I2-I1-M-ND2* (Figure 5). Although tRNA rearrangement is a relatively common occurrence, the presence of extra tRNAs is a rare event. In Reduviidae, this phenomenon has only been reported in *Brontostoma colossus*, in which an additional copy of the *trnR* is brought out by the TDRL mechanism [26]. Interestingly, the two copies of *trnR* in *B. colossus* are absolutely identical, whereas in *R. tenebrosus*, *trnI2* is not merely a copy of *trnI1*; instead, the extra *trnI2* is highly similar to *trnM* (Figure 2).

In order to further probe whether the gene rearrangement of *R. tenebrosus* is a common feature in the subfamily Reduviinae, we amplified and sequenced two fragments between the CR and *ND2* from two additional species of Reduviinae—*Reduvius gregorgi* and *Acanthaspis cincticrus* (GenBank accession numbers: KX241473 and KX241472). The results show that both fragments retain the ancestral arrangement (CR-*I-Q-M-ND2*). This suggests that genome rearrangement is not widely distributed within Reduviinae.

### 2.3. Mechanisms Account for the Gene Rearrangements in Reduvius tenebrosus mt Genome

According to the TDRL model, duplication of part of the mt genome is caused by slipped-strand mispairing or erroneous identification of the origin of light-strand replication. As part of this process, one gene from each of the duplicated gene pairs is randomly eliminated, at which point it becomes a pseudo gene. Pseudo genes then accumulate additional mutations and become NCRs. It is also possible that pseudo genes may become lost, which creates a novel gene order [3,18]. As mentioned above, the presence and location of pseudo-*trnI*, pseudo-*ND2*, pseudo-CR and *trnI2* may shed light on whether a novel gene order can be explained by the TDRL model.

**Figure 6:**
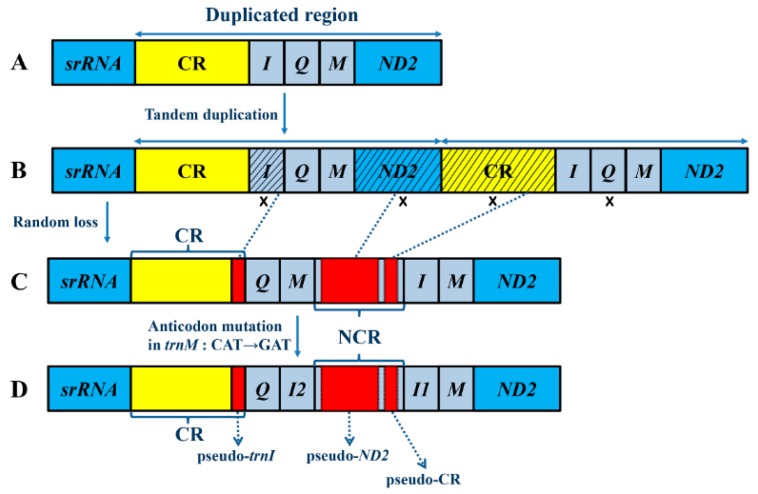
The hypothetical process of gene rearrangement in the model of tandem duplication/random loss and duplication/anticodon mutation: (**A**) the ancestral gene order in insects; (**B**) the tandem duplication of CR/*trnI*/*trnQ*/*trnM*/*ND2*; (**C**) the random loss of the duplicated *trnI*, *ND2*, CR and *trnQ*; and (**D**) the anticodon mutation in *trnM* which changes *trnM* (CAT) to *trnI2* (GAT). “x” indicates the random loss of the duplicated genes. Shaded areas indicate the genes or regions that are partially lost. Dotted arrows connect the original genes to the rearranged products. Random lost genes and their corresponding pseudo genes are indicated by blue dotted lines. CR is the abbreviation for control region and NCR is the abbreviation for non-coding region.

The steps of the TDRL are as follows: First, the gene *CR*-*trnI*-*trnQ-trnM*-*ND2* (Figure 6A) is tandemly duplicated and generates two sets of the same gene cluster (*CR*-*trnI*-*trnQ-trnM*-*ND2-CR*-*trnI*-*trnQ-trnM*-*ND2*) (Figure 6B). Then, a new order *CR*-*trnQ-trnM*-*trnI*-*trnM*-*ND2* is generated after one gene form each of the five duplicated gene pairs is randomly eliminated and becomes a pseudo gene. The pseudo genes then accumulate additional mutations and become NCRs or even be lost (see Figure 6C). The presence of pseudo-*trnI* in the CR, as well as the presence of pseudo-*ND2* and pseudo-*CR* in the long NCR between *trnI2* and *trnI1*, combined with the significant similarities between *trnI2* and *trnM*, all support our speculated steps of gene duplication and loss in gene rearrangements. Meanwhile, the case that gene rearrangement between CR and *ND2* only occurs in *R. tenebrosus* among the 13 sequenced assassin bug mt genomes and two fragments between CR and *ND2* of two Reduviinae species also suggest a recent origin of duplication events as well.

Obviously, the novel gene order *CR*-*trnQ-trnI2*-*trnI1*-*trnM*-*ND2* cannot be entirely explained by the TDRL mechanism, because this model only generates the order *CR*-*trnQ-trnM*-*trnI*-*trnM*-*ND2*. As such, we use the duplication/anticodon mutation mechanism [23] to further explain the transformation from *trnM* to *trnI2* (Figure 6D), in which *trnI2* remolds from the duplicated *trnM* through an anticodon switch (CAT to GAT) and two other site mutations. We also use the tRNA duplication/anticodon mutation model to explain the occurrence of the duplicated *trnW* in *Bothriometopus* which is derived from *trnE* via anticodon mutations (UUC to UCA) [43]. This duplication of a tRNA gene, followed by an anticodon change, provides an example of the types of events that could underlie shifts in the genetic code (which occur often during mtDNA evolution [22,44]).

**Figure 7:**
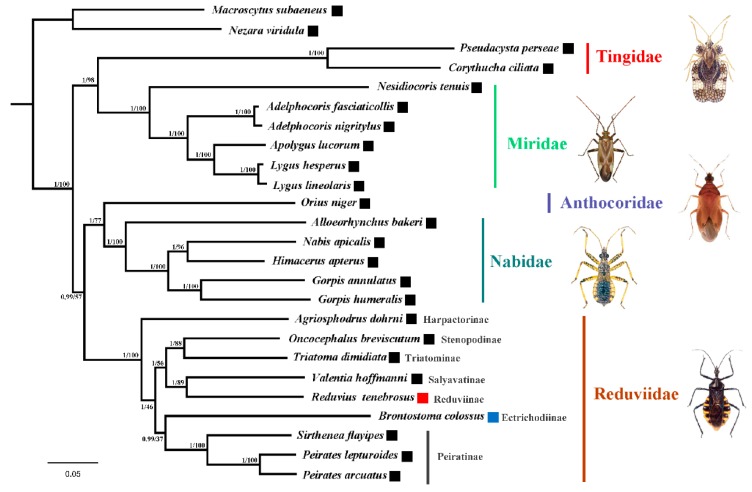
The phylogenetic relationships among the seven reduviid subfamilies and five cimicomorphan families inferred from the mt genome sequences. Numbers close to the branching points are Bayesian posterior probabilities and maximum likelihood (ML) bootstrap support values. The black boxes refer to species without duplicated tRNAs. Red boxes indicate duplicate tRNA derived from the tRNA duplication/anticodon mutation model. Blue boxes represent duplicated tRNA caused by the tandem duplication/random loss model.

The presence of extra tRNAs is a rare event in the family Reduviidae. Extra tRNAs have only been found in *B. colossus* (the subfamily Ectrichodiinae) with a duplicated *trnR* derived from the TDRL mechanism [26] and in *R. tenebrosus* (the subfamily Reduviinae) with an extra *trnI2* generated by the tRNA duplication/anticodon mutation model. Phylogenetic analyses showed almost identical topologies for the relationships among the five cimicomorphan families (Figure 7). Within Reduviidae, the sister-group relationship between Ectrichodiinae and Piratinae and a close relationship between Reduviinae and Salyavatinae is supported. This is consistent with previous analyses based on mitochondrial and nuclear ribosomal genes [45]. These results indicate that the duplicated tRNA gene in the mitochondrial genome has evolved independently at least two times within Reduviidae.

Mitochondrial (mt) genome rearrangements in many other taxa occur frequently in the region between the CR and *ND2*, such as the rearrangement from *trnI-trnQ-trnM* to *trnM-trnI-trnQ* in Formicidae (Hymenoptera) [46] and Ditrysia (Lepidoptera) [47], and from *trnI-trnQ-trnM* to *trnQ-trnI-trnM* in Aradidae [41,42]. In Hymenoptera and Lepidoptera, identical rearrangement occurs independently, with numerous possible combinations of duplications and deletions. Many studies have noted that tandem duplications and gene deletions may be subject to mechanistic constraints, so that genes flanking the origins of strand replication (e.g., the CR) are more likely to be duplicated. This forms a “hot spot” for gene rearrangement that makes convergent gene order rearrangement more probable [3]. For the sake of “hot spot”, local or even fully homoplastic rearrangements may be more common than previously thought, therefore, when genome orders are employed as important molecular signatures, we should make sure the data are considered in a phylogenetic and systematic context. Moreover, we should make comparisons of complete gene orders rather than focusing solely on local arrangements [48].

## 3. Materials and Methods

### 3.1. Specimen Collection and DNA Extraction

We collected specimens of *R. tenebrosus* from the Maolan National Nature Reserve, Guizhou, China. The specimens were stored in 100% ethanol at −20 °C at the Entomological Museum of China Agricultural University (Beijing, China). We extracted total DNA from the thoracic muscle of one adult specimen using the DNeasy DNA Extraction kit (Qiagen, Hilden, Germany).

### 3.2. Polymerase Chain Reaction (PCR) Amplification and Sequencing

The complete mt genome was amplified by polymerase chain reaction (PCR) in overlapping fragments with 13 universal insect mt primers [49], and eight species-specific primers designed from sequenced fragments. We list all the primers used in this study in Table S7. We conducted PCR and sequencing reactions following the guidelines set forth by Li *et al.* [29,50].

### 3.3. Genome Annotation and Sequence Analysis

We assembled raw sequences into contigs using BioEdit version 7.0.5.3 [51]. We annotated the complete mt genome sequence using the MITOS webserver with the invertebrate mitochondrial code [52]. We re-confirmed the tRNA genes by using tRNAscan-SE Search Server version 1.21 [53]. tRNA genes that could not be identified by using the MITOS webserver [52] and tRNAscan-SE were determined by using sequence similarity comparison with tRNA genes of other true bugs. Finally, we manually checked all automatic annotations. We deposited the complete mt genome of *R. tenebrosus* in the GenBank database under the accession number KC887529.

We computed nucleotide composition and codon usage by using MEGA 6.0 program [54]. We used AT-skew [(A − T)/(A + T)] and GC-skew [(G − C)/(G + C)] to measure nucleotide compositional differences between genes [55]. Synonymous codon composition is represented by relative synonymous codon usage (RSCU) [56]. We used the chi-square test (with 5% significance level) to evaluate the statistical significance of our results. We assumed the relative frequency of ATT *vs.* ATC to be equal from species to species as a null hypothesis. We evaluated the deviation of the relative frequency in a species (from the average value among the 13 assassin bug species) with the chi-square test [32].

### 3.4. Phylogenetic Analysis

We used sequences from 13 PCGs and two rRNAs from 23 cimicomorphan insects, as well as two outgroup species from Pentatomomorpha for phylogenetic analyses. Details of species we used in this study are shown in Table S8.

We individually aligned each PCG based on codon-based multiple alignments using the MAFFT algorithm within the TranslatorX online platform (with the L-INS-i strategy and default setting) [57]. We aligned the sequences of rRNAs using the MAFFT 7.0 online server (with the G-INS-i strategy) [58]. We concentrated the alignments of the individual genes into a large dataset by removing the third codon position of the PCGs. We analyzed the dataset under Bayesian (BI) and maximum likelihood (ML) frameworks using MrBayes 3.2.3 (with the GTR + I + G model) [59] and RAxML-HPC2 8.1.11 (with the GTR + I + G model) [60] respectively. We created separate partitions for each gene in the dataset. Bootstrap ML analyses with 1000 replicates were performed with the fast ML method implemented in RAxML using the GTRGAMMA model [61]. For MrBayes, two simultaneous runs of 10 million generations were conducted for the datasets and trees were sampled every 1000 generations, with the first 25% discarded as burn-in.

## 4. Conclusions

In this study, we discovered and described the duplication, remolding and rearrangement of tRNA genes in the complete mt genome of the assassin bug *R. tenebrosus*. Pseudo genes and rearranged tRNA genes in the hotspot region of gene rearrangement (between the control region and *ND2*) indicate that the main mechanism underlying the rearrangement of the *R. tenebrosus* mt genome is the TDRL model. Additionally, the tRNA duplication/anticodon mutation mechanism further explains the presence of *trnI2*, which is remolded from a duplicated *trnM* in the TDRL process (through an anticodon mutation of CAT to GAT). However, it is unclear whether the two events proceed simultaneously, and if the remolded tRNAs are functional. The remolding of duplicated tRNA genes is a rare event in insect mt genomes and this novel mt gene rearrangement in assassin bugs improves our understanding of the evolution of mt gene arrangements in insects.

## Figures and Tables

**Table 1:** Mitochondrial gene rearrangements in true bug mt genomes.

Classification	Species	Rearrangement	GenBank Accession	Reference
Aradidae	*Brachyrhynchus hsiaoi*	*I-Q*→*Q-I*	HQ441232	[41]
Aradidae	*Aradacanthia heissi*	*I-Q*→*Q-I*; *W-C*→*C-W*	HQ441233	[42]
Aradidae	*Neuroctenus parus*	*I-Q*→*Q-I*	EU427340	[40]
Pyrrhocoridae	*Dysdercus Cingulatus*	*T-P*→*P-T*	NC_012421	[40]
Largidae	*Physopelta gutta*	*T-P*→*P-T*	NC_012432	[40]
Enicocephalidae	*Stenopirates* sp.	*T-P-ND6-CytB-S2-ND1-L2-lrRNA-V-srRNA-*CR→*CytB-S2-*CR*-lrRNA-V-srRNA-ND1-L2-P-T-ND6*	NC_016017	[17]
Reduviidae	*Brontostoma Colossus*	*A-R-N-S1*→*R-A-R-N-S1*	KM044501	[26]
Reduviidae	*Reduvius Tenebrosus*	*I-Q-M*→*Q-I2-I1-M*	KC887529	Present study

## Supplementary Materials

Supplementary materials can be found at http://www.mdpi.com/1422-0067/17/6/951/s1.
