# Supplementary material for: Duplication and Remolding of tRNA Genes in the Mitochondrial Genome of Reduvius tenebrosus (Hemiptera: Reduviidae)
Source: Int J Mol Sci. 2016 Jun 16;17(6):951. doi: 10.3390/ijms17060951 (PMC4926484; doi:10.3390/ijms17060951)
Supplement: Supplementary file 1 [file ijms-17-00951-s001.pdf]

Supplementary Materials: Duplication and Remolding of tRNA Genes in the Mitochondrial Genome of *Reduvius tenebrosus* (Hemiptera: Reduviidae)

Pei Jiang, Hu Li, Fan Song, Yao Cai, Jianyun Wang, Jinpeng Liu and Wanzhi Cai

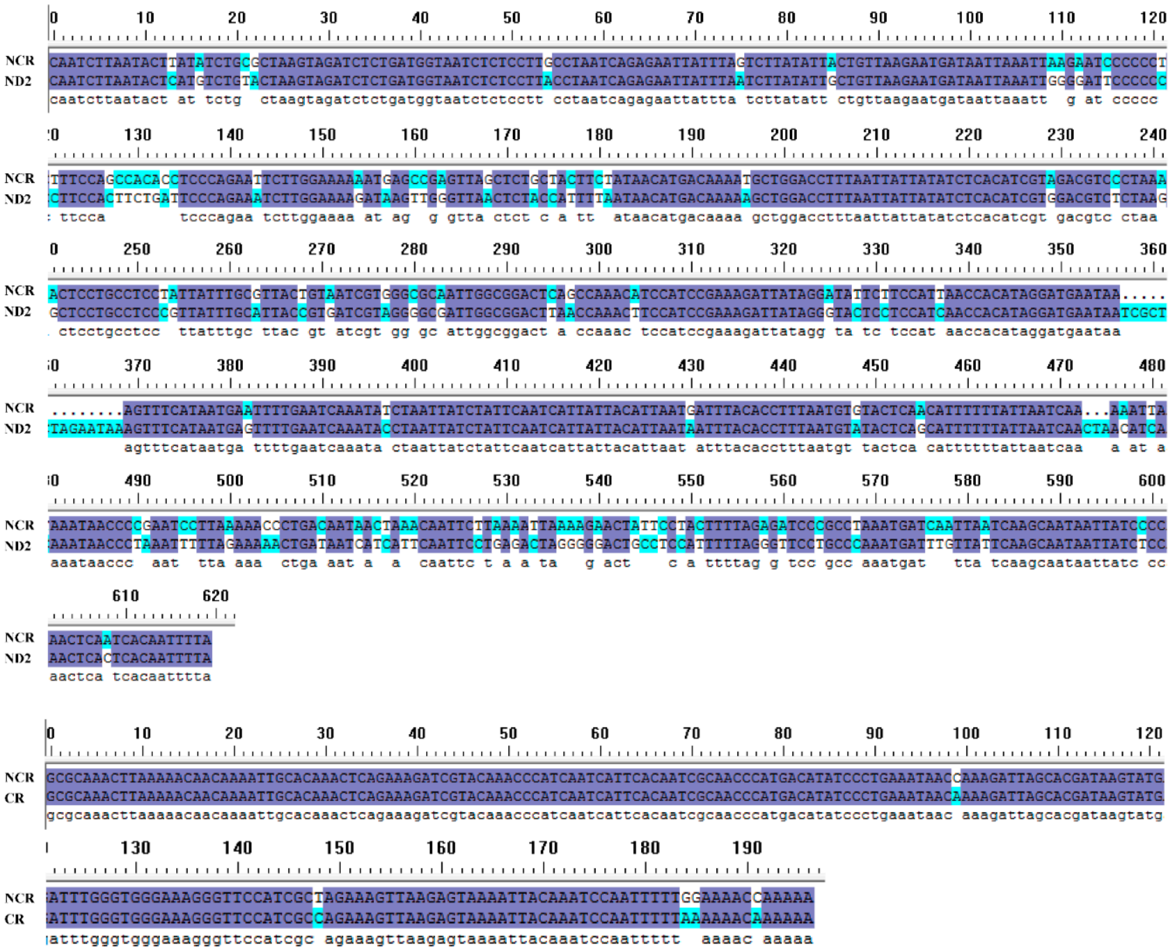

Figure S1. Sequence matching between the non-coding regions (NCR) parts and *ND2* and control region (CR). The identical bases between two sequences are in navy blue and the white and sky blue ones indicate different bases between them.

Table S1. Organization of *Reduvius tenebrosus* mitochondrial (mt) genome. "F" indicates genes encoded on the majority strand and "R" indicates genes annotated on the minority strand. "T-" stands for the incomplete stop codon (a single T).

Gene	Direction	Location		Size	Anticodon	Codon Start	Codon Stop	Intergenic Nucleotides
trnI1	F	1	64	64	GAT			0
trnM	F	107	175	69	CAT			42
ND2	F	176	1177	1002		ATC	TAA	0
trnW	F	1176	1240	65	TCA			−2
trnC	R	1233	1294	62	GCA			−8
trnY	R	1303	1368	66	GTA			8
COI	F	1372	2905	1534		ATG	T-	3

Table S1. *Cont.*

Gene	Direction	Location		Size	Anticodon	Codon Start	Codon Stop	Intergenic Nucleotides
trnL2	F	2906	2970	65	TAA			0
COII	F	2971	3654	684		ATT	TAA	0
trnK	F	3672	3741	70	CTT			17
trnD	F	3742	3804	63	GTC			0
ATP8	F	3805	3963	159		ATT	TAA	0
ATP6	F	3957	4640	684		ATG	TAG	−7
COIII	F	4627	5413	787		ATG	T-	−14
trnG	F	5414	5475	62	TCC			0
ND3	F	5476	5829	354		ATA	TAG	0
trnA	F	5829	5888	60	TGC			−1
trnR	F	5891	5954	64	TCG			2
trnN	F	6058	6124	67	GTT			103
trnS1	F	6124	6192	69	GCT			−1
trnE	F	6194	6258	65	TTC			1
trnF	R	6257	6323	67	GAA			−2
ND5	R	6324	8034	1711		ATT	T-	0
trnH	R	8035	8096	62	GTG			0
ND4	R	8097	9423	1327		ATG	T-	0
ND4L	R	9417	9713	297		GTG	TAA	−7
trnT	F	9716	9776	61	TGT			2
trnP	R	9777	9842	66	TGG			0
ND6	F	9845	10,345	501		ATT	TAA	2
CytB	F	10,345	11,481	1137		ATG	TAA	−1
trnS2	F	11,480	11,550	71	TGA			−2
ND1	R	11,696	12,613	918		GTG	TAG	145
trnL1	R	12,614	12,678	65	TAG			0
lrRNA	R	12,679	13,933	1255				0
trnV	R	13,934	14,001	68	TAC			0
srRNA	R	14,002	14,785	784				0
control region	F	14,786	16,036	1251				0
trnQ	R	16,037	16,105	69	TTG			0
trnI2	F	16,105	16,173	69	GAT			−1
NCR	F	16,174	17,090	917				0

Table S2. Base composition in *R. tenebrosus* mt genome.

Feature	Total	%T	%C	%A	%G	%A+T	%G+C	AT-Skew	GC-Skew
Whole genome	17,090	27.5	20.4	39.7	12.5	67.2	32.8	0.18	−0.24
Protein-coding genes	11,064	38.2	17.8	27.6	16.5	65.8	34.2	−0.16	−0.04
First codon position	3688	31.9	15.9	31.5	20.7	63.4	36.6	−0.01	0.13
Second codon position	3688	45.9	19.3	18.8	16.1	64.7	35.3	−0.42	−0.09
Third codon position	3688	36.7	18.1	32.5	12.6	69.2	30.8	−0.06	−0.18
Protein-coding genes-J	6819	30.6	22	32.8	14.6	63.4	36.6	0.03	−0.20
First codon postion	2273	24.3	19.4	35.5	20.7	59.8	40.2	0.19	0.03
Second codon position	2273	43.6	21.4	20.1	15	63.7	36.3	−0.37	−0.18
Third codon position	2273	23.9	25	42.8	8.2	66.7	33.3	0.28	−0.51
Protein-coding genes-N	4245	50.3	11.1	19.2	19.4	69.5	30.5	−0.45	0.27
First codon position	1415	44.1	10.2	25.1	20.6	69.2	30.8	−0.27	0.34
Second codon postion	1415	49.5	15.9	16.7	17.9	66.2	33.8	−0.50	0.06
Third codon position	1415	57.3	7.1	15.9	19.7	73.2	26.8	−0.57	0.47
Control region	1251	28.9	21.7	37.2	12.2	66.1	33.9	0.13	-0.28

Table S3. Structural features and codon usage for isoleucine (Ile) of sequenced assassin bug mt genomes.

Subfamily	Species	Rearragement	Length (bp)	AT %	Codons for Ile			GenBank Accession
					ATT	ATC	ATT + ATC	
Triatominae	*Triatoma dimidiata*	none	17,019	69.5%	281 *	81 *	362	NC_002609
Salyavatinae	*Valentia hoffmanni*	none	15,625	73.7%	325	41	366	NC_012823
Harpactorinae	*Agriosphodrus dohrni*	none	16,470	72.2%	313	48	361	NC_015842
Ectrichodiinae	*Brontostoma colossus*	*A-R-N→R-A-R-N*	16,625	73.4%	328	56	384	NC_024745
Peiratinae	*Sirthenea flavipes*	none	15,961	71.8%	328	51	379	NC_020143
Peiratinae	*Peirates arcuatus*	none	16,176	71.3%	343 *	42 *	385	NC_024264
Peiratinae	*Peirates fulvescens*	none	15,702	71.9%	318	53	371	KF913537
Peiratinae	*Peirates atromaculatus*	none	16,151	71.0%	309	61	370	KF913539
Peiratinae	*Peirates turpis*	none	15,703	72.0%	312	58	370	KF913540
Peiratinae	*Peirates lepturoides*	none	15,932	72.4%	329	59	388	KF913541
Stenopodinae	*Oncocephalus breviscutum*	none	15,948	74.4%	367 *	24 *	391	NC_022816
Tribelocephalinae	*Opistoplatys* sp.	none	15,615	75.6%	383 *	38 *	421	KC887533
Reduviinae	*Reduvius tenebrosus*	*I-Q-M→Q-I-I-M*	17,090	67.2%	251 *	114 *	365	present study

Values with an asterisk signify that relative frequency of codons for ATT *vs.* ATC is significantly ($p < 0.05$) different from the averaged among 13 assassin bugs.

Table S4. Codon usage of protein-coding genes in the *R. tenebrosus* mt genome.

Amino Acid	Codon	N	RSCU	N+	RSCU	N−	RSCU
Phe (F)	**UUU**	**235**	**1.41**	78	0.97	**157**	**1.83**
	UUC	98	0.59	**83**	**1.03**	15	0.17
Leu (L)	**UUA**	**186**	**2.08**	87	1.83	**99**	**2.37**
	UUG	103	1.15	12	0.25	91	2.18
	CUU	76	0.85	32	0.67	44	1.05
	CUC	41	0.46	39	0.82	2	0.05
	CUA	104	1.16	**94**	**1.97**	10	0.24
	CUG	27	0.30	22	0.46	5	0.12
Ile (I)	**AUU**	**251**	**1.38**	**155**	**1.21**	**96**	**1.76**
	AUC	114	0.62	101	0.79	13	0.24
Met (M)	**AUA**	**184**	**1.47**	**147**	**1.74**	37	0.91
	AUG	66	0.53	22	0.26	**44**	**1.09**
Val(V)	GUU	89	1.72	30	1.01	**59**	**2.68**
	GUC	24	0.46	17	0.57	7	0.32
	**GUA**	**66**	**1.28**	**57**	**1.92**	9	0.41
	GUG	28	0.54	15	0.50	13	0.59
Ser (S)	**UCU**	**106**	**2.25**	23	0.88	**83**	**3.88**
	UCC	48	1.02	39	1.50	9	0.42
	UCA	71	1.51	**63**	**2.42**	8	0.37
	UCG	7	0.15	2	0.08	5	0.23
Pro (P)	**CCU**	**60**	**1.71**	31	1.16	**29**	**3.52**
	CCC	39	1.11	**36**	**1.35**	3	0.36
	CCA	36	1.03	35	1.31	1	0.12
	CCG	5	0.14	5	0.19	0	0.00
Thr (T)	**ACU**	**69**	**1.56**	35	1.01	**34**	**3.58**
	ACC	24	0.54	23	0.66	1	0.11
	ACA	74	1.67	**73**	**2.10**	1	0.11
	ACG	10	0.23	8	0.23	2	0.21
Ala (A)	GCU	49	1.21	16	0.57	**33**	**2.69**
	GCC	38	0.94	31	1.10	7	0.57
	**GCA**	**65**	**1.60**	**62**	**2.19**	3	0.24
	GCG	10	0.25	4	0.14	6	0.49
Tyr (Y)	**UAU**	**108**	**1.32**	31	0.81	**77**	**1.77**
	UAC	56	0.68	**46**	**1.19**	10	0.23

Table S4. *Cont.*

Amino Acid	Codon	N	RSCU	N+	RSCU	N−	RSCU
Stop (*)	UAA	6					
	UAG	3					
His (H)	**CAU**	**37**	**0.99**	26	0.85	**11**	**1.57**
	<u>CAC</u>	38	1.01	**35**	**1.15**	3	0.43
Gln (Q)	**<u>CAA</u>**	**44**	**1.28**	**41**	**1.58**	3	0.35
	CAG	25	0.72	11	0.42	**14**	**1.65**
Asn (N)	**AAU**	**71**	**1.08**	35	0.75	**36**	**1.85**
	<u>AAC</u>	61	0.92	**58**	**1.25**	3	0.15
Lys (K)	**AAA**	**54**	**1.19**	**51**	**1.48**	3	0.27
	<u>AAG</u>	37	0.81	18	0.52	**19**	**1.73**
Asp (D)	**GAU**	**54**	**1.38**	**26**	**1.11**	**28**	**1.81**
	<u>GAC</u>	24	0.62	21	0.89	3	0.19
Glu (E)	**<u>GAA</u>**	**53**	**1.28**	**47**	**1.65**	6	0.46
	GAG	30	0.72	10	0.35	**20**	**1.54**
Cys (C)	**UGU**	**38**	**1.36**	5	0.56	**33**	**1.74**
	<u>UGC</u>	18	0.64	**13**	**1.44**	5	0.26
Trp (W)	**<u>UGA</u>**	**74**	**1.44**	**58**	**1.63**	**16**	**1.00**
	UGG	29	0.56	13	0.37	**16**	**1.00**
Arg (R)	CGU	17	0.68	3	0.34	**14**	**2.80**
	CGC	4	0.16	3	0.34	1	0.20
	**<u>CGA</u>**	**26**	**1.03**	**24**	**2.74**	2	0.40
	CGG	8	0.32	5	0.57	3	0.60
Ser (S)	AGU	36	0.76	9	0.35	27	1.26
	<u>AGC</u>	15	0.32	9	0.35	6	0.28
	AGA	82	3.26	61	2.35	21	0.98
	AGG	14	0.56	2	0.08	12	0.56
Gly (G)	GGU	59	1.02	9	0.27	**50**	**2.06**
	GGC	27	0.47	15	0.44	12	0.49
	**<u>GGA</u>**	**79**	**1.36**	**73**	**2.16**	6	0.25
	GGG	67	1.16	38	1.13	29	1.20

N, the number of codons used in protein-coding genes; RSCU, relative synonymous codon usage. Values in bold type stand for the most commonly used codon for amino acid. Underlined stand for the cognate codon of tRNA for each amino acid.

Table S5. Codon usage of protein-coding genes in 13 sequenced assassin bug mt genomes.

Species	Phe			Leu							Ile			Met			Val				
	**UUU**	**UUC**	**%**	**UUA**	**UUG**	**CUU**	**CUC**	**CUA**	**CUG**	**%**	**AUU**	**AUC**	**%**	**AUA**	**AUG**	**%**	**GUU**	**GUC**	**GUA**	**GUG**	**%**
Triatoma dimidiata	250	85	0.25	219	95	89	31	81	10	0.26	281	81	0.22	193	58	0.23	113	21	76	19	0.17
Valentia hoffmanni	292	55	0.16	323	52	82	9	53	7	0.13	325	41	0.11	250	31	0.11	92	9	86	9	0.09
Agriosphodrus dohrni	282	71	0.20	298	51	78	22	55	17	0.17	313	48	0.13	222	39	0.15	83	19	82	19	0.19
Brontostoma colossus	276	82	0.23	295	74	54	10	68	10	0.18	328	56	0.15	247	43	0.15	90	13	58	7	0.12
Sirthenea flavipes	283	62	0.18	264	68	67	17	82	11	0.19	328	51	0.13	217	64	0.23	91	20	78	16	0.18
Peirates arcuatus	278	64	0.19	275	56	76	15	68	18	0.18	343	42	0.11	209	50	0.19	91	15	78	15	0.15
Peirates fulvescens	269	75	0.22	331	61	56	22	46	10	0.18	318	53	0.14	216	49	0.18	106	11	74	17	0.13
Peirates atromaculatus	276	69	0.20	330	60	60	17	50	9	0.16	309	61	0.16	214	51	0.19	102	15	75	17	0.15
Peirates turpis	273	71	0.21	332	60	60	17	46	12	0.17	312	58	0.16	214	58	0.21	103	15	74	17	0.15
Peirates lepturoides	289	54	0.16	315	49	75	12	54	9	0.14	329	59	0.15	221	47	0.18	106	12	71	7	0.10
Oncocephalus breviscutum	287	61	0.18	325	45	84	9	35	6	0.12	367	24	0.06	251	22	0.08	94	5	74	13	0.10
Opistoplatys sp.	313	43	0.12	367	38	57	10	33	2	0.10	383	38	0.09	255	29	0.10	71	11	68	11	0.14
Reduvius tenebrosus	235	98	0.29	186	103	76	41	104	27	0.32	251	114	0.31	184	66	0.26	89	24	66	28	0.25
Average	277	68	0.20	297	62	70	18	60	11	0.18	322	56	0.15	223	47	0.17	95	15	74	15	0.15

Species	Ser					Pro					Thr					Ala					Tyr		
	**UCU**	**UCC**	**UCA**	**UCG**	**%**	**CCU**	**CCC**	**CCA**	**CCG**	**%**	**ACU**	**ACC**	**ACA**	**ACG**	**%**	**GCU**	**GCC**	**GCA**	**GCG**	**%**	**UAU**	**UAC**	**%**
T. dimidiata	130	26	76	3	0.12	67	21	41	4	0.19	77	37	69	5	0.22	60	35	61	4	0.24	130	37	0.22
V. hoffmanni	108	28	101	2	0.13	69	19	45	0	0.14	76	16	92	2	0.10	65	15	60	2	0.12	138	22	0.14
A. dohrni	110	22	88	2	0.11	54	22	47	2	0.19	97	25	80	5	0.14	56	23	67	5	0.19	137	38	0.22
B. colossus	108	22	85	9	0.14	63	20	51	5	0.18	61	23	101	7	0.16	48	12	55	3	0.13	145	29	0.17
S. flavipes	129	18	77	6	0.10	53	25	53	8	0.24	76	15	89	6	0.11	66	20	54	4	0.17	136	33	0.20
P. arcuatus	117	27	77	8	0.15	52	33	50	4	0.27	79	21	87	5	0.14	46	29	68	3	0.22	139	28	0.17
P.fulvescens	121	18	83	8	0.11	63	25	50	3	0.20	65	22	93	3	0.14	42	21	79	5	0.18	138	24	0.15
P. atromaculatus	123	18	84	5	0.10	63	26	53	3	0.20	66	20	96	1	0.11	41	21	78	5	0.18	140	23	0.14
P. turpis	124	16	85	6	0.10	62	27	52	3	0.21	66	20	95	2	0.12	42	19	78	5	0.17	140	23	0.14
P. lepturoides	123	12	94	2	0.06	74	17	50	3	0.14	77	16	84	1	0.10	55	19	82	4	0.14	140	24	0.15
O. breviscutum	125	20	86	6	0.11	70	8	56	2	0.07	71	17	91	5	0.12	66	15	49	6	0.15	161	11	0.06
Opistoplatys sp.	105	23	80	4	0.13	71	16	41	7	0.17	77	22	58	5	0.17	53	26	54	4	0.22	171	14	0.08
R. tenebrosus	106	48	71	7	0.24	60	39	36	5	0.31	69	24	74	10	0.19	49	38	65	10	0.30	108	56	0.34
Average	118	23	84	5	0.12	63	23	48	4	0.19	74	21	85	4	0.14	53	23	65	5	0.19	140	28	0.17

Species	His			Gln			Asn			Lys			Asp			Glu			Cys		
	CAU	CAC	%	CAA	CAG	%	AAU	AAC	%	AAA	AAG	%	GAU	GAC	%	GAA	GAG	%	UGU	UGC	%
T. dimidiata	49	30	0.38	48	9	0.16	105	44	0.30	50	35	0.41	48	25	0.34	61	26	0.30	41	14	0.25
V. hoffmanni	58	21	0.27	57	5	0.08	130	29	0.18	69	24	0.26	54	13	0.19	63	20	0.24	47	10	0.18
A. dohrni	55	24	0.30	47	16	0.25	130	40	0.24	69	39	0.36	54	21	0.28	70	21	0.23	53	1	0.02
B. colossus	55	22	0.29	47	9	0.16	128	38	0.23	92	15	0.14	54	15	0.22	58	12	0.17	56	6	0.10
S. flavipes	46	31	0.40	47	10	0.18	115	42	0.27	68	32	0.32	52	23	0.31	68	20	0.23	34	18	0.35
P. arcuatus	59	17	0.22	47	11	0.19	123	28	0.19	65	24	0.27	56	19	0.25	75	16	0.18	49	12	0.20
P.fulvescens	49	30	0.38	44	10	0.19	117	34	0.23	75	25	0.25	57	18	0.24	72	16	0.18	52	8	0.13
P. atromaculatus	47	30	0.39	47	8	0.15	117	34	0.23	71	29	0.29	56	19	0.25	72	19	0.21	51	9	0.15
P. turpis	48	29	0.38	47	8	0.15	117	34	0.23	71	31	0.30	57	18	0.24	72	17	0.19	51	9	0.15
P. lepturoides	51	27	0.35	47	8	0.15	125	26	0.17	59	39	0.40	64	12	0.16	71	17	0.19	46	12	0.21
O. breviscutum	57	17	0.23	55	9	0.14	144	17	0.11	84	17	0.17	62	6	0.09	81	12	0.13	46	6	0.12
Opistoplatys sp.	57	13	0.19	56	8	0.13	169	20	0.11	81	15	0.16	59	8	0.12	71	15	0.17	46	6	0.12
R. tenebrosus	37	38	0.51	44	25	0.36	71	61	0.46	54	37	0.41	54	24	0.31	53	30	0.36	38	18	0.32
Average	51	25	0.33	49	10	0.18	122	34	0.22	70	28	0.29	56	17	0.23	68	19	0.21	47	10	0.17

Species	Trp			Arg					Ser					Gly				
	UGA	UGG	%	CGU	CGC	CGA	CGG	%	AGU	AGC	AGA	AGG	%	GGU	GGC	GGA	GGG	%
T. dimidiata	77	23	0.23	18	1	33	4	0.09	27	7	92	9	0.12	72	20	109	27	0.21
V. hoffmanni	90	11	0.11	19	1	29	4	0.09	28	10	91	4	0.11	67	8	115	33	0.18
A. dohrni	83	22	0.21	16	2	32	2	0.08	35	9	82	11	0.15	68	14	76	45	0.29
B. colossus	78	16	0.17	14	4	25	6	0.20	34	9	72	8	0.14	69	19	111	31	0.22
S. flavipes	88	14	0.14	20	1	30	3	0.07	33	8	85	3	0.09	74	7	106	34	0.19
P. arcuatus	85	18	0.17	19	5	28	2	0.13	28	6	92	14	0.14	66	9	115	39	0.21
P.fulvescens	89	16	0.15	16	2	32	4	0.11	33	4	93	5	0.07	81	12	96	34	0.21
P. atromaculatus	86	17	0.17	16	2	31	5	0.13	31	6	94	5	0.08	80	12	97	34	0.21
P. turpis	86	17	0.17	16	2	31	5	0.13	31	6	94	3	0.07	78	13	98	33	0.21
P. lepturoides	90	13	0.13	15	2	32	5	0.13	31	5	89	7	0.09	79	10	102	34	0.20
O. breviscutum	91	12	0.12	19	1	30	5	0.11	35	4	87	3	0.05	88	0	120	19	0.08
Opistoplatys sp.	81	17	0.17	25	2	21	5	0.13	33	4	72	4	0.07	63	13	110	34	0.21
R. tenebrosus	74	29	0.28	17	4	26	8	0.22	36	15	82	14	0.20	59	27	79	67	0.41
Average	84	17	0.17	18	2	29	4	0.12	32	7	87	7	0.11	73	13	103	36	0.22

%: The ratios of codons ending in G or C *vs.* total codons of each amino acid.

Table S6. Start and stop codons of protein-coding genes in sequenced assassin bug mt genomes.

Gene	Start/Stop Codon												
	T. dimidiata	***V. hoffmanni***	***A. dohrni***	***S. flavipes***	***B. colossus***	***P. arcuatus***	***O. breviscutum***	***Opistoplatys* sp.**	***R. tenebrosus***	***P. fulvescens***	***P. atromaculatus***	***P. turpis***	***P. lepturoides***
ND2	ATC/TAG	ATG/TAA	ATT/TAA	ATT/TAA	ATT/T-	ATG/TAA	ATT/T-	ATT/TAA	ATC/TAA	ATG/TAA	ATG/TAA	ATG/TAA	ATG/TAA
COI	ATG/T-	ATG/T-	ATG/T-	ATG/TAA	ATG/T-	ATG/T-	ATG/T-	ATG/TAA	ATG/T-	ATG/T-	ATG/T-	ATG/T-	ATG/T-
COII	ATA/T-	ATA/T-	ATC/TAA	ATA/TAA	ATT/T-	ATC/T-	ATG/T-	ATA/T-	ATT/TAA	ATC/T-	ATC/T-	ATC/T-	ATC/T-
ATP8	ATA/TAA	ATC/TAA	ATT/TAA	ATA/TAA	ATT/TAA	ATT/TAA	ATA/TAA	ATA/TAA	ATT/TAA	ATA/TAA	ATA/TAA	ATA/TAA	ATT/TAA
ATP6	ATG/TAA	ATG/TAG	ATG/TAA	ATG/TAG	ATG/TAA	ATG/TAA	ATG/TAG	ATG/TAA	ATG/TAG	ATG/TAG	ATG/TAG	ATG/TAG	ATG/TAG
COIII	ATG/TA-	ATG/T-	ATG/T-	ATG/T-	ATG/TAA	ATG/T-	ATG/T-	ATG/T-	ATG/T-	ATG/T-	ATG/T-	ATG/T-	ATG/T-
ND3	ATA/TA-	ATT/T-	ATA/TAA	ATA/T-	ATA/TAA	ATT/TAA	ATT/TA-	ATT/TAA	ATA/TAG	ATT/T-	ATT/T-	ATT/T-	ATT/T-
ND5	GTG/TA-	ATT/T-	ATG/T-	ATT/T-	ATT/T	ATT/TAA	ATT/T-	ATT/T-	ATT/T-	ATT/T-	ATT/T-	ATT/T-	ATT/T-
ND4	ATG/TAA	ATG/TAA	ATG/TAA	ATG/TAA	ATG/TAA	ATG/TAA	ATG/TAG	ATG/TAA	ATG/T-	ATG/TAA	ATG/TAA	ATG/TAA	ATG/TAA
ND4L	ATG/TAA	ATT/TAA	ATG/TAA	GTG/TAA	ATT/TAA	ATG/TAA	GTG/TAA	ATT/TAG	GTG/TAA	ATG/TAA	ATG/TAA	ATG/TAA	ATG/TAA
ND6	ATA/TAA	ATA/TAA	ATG/TAA	ATT/TAA	ATA/TAA	ATA/TAA	ATG/TAA	ATA/TAA	ATT/TAA	ATT/TAA	ATT/TAA	ATT/TAA	ATC/TAA
CytB	ATG/T-	ATG/TAG	ATG/TAA	ATG/TAG	ATG/T-	ATG/TAA	ATA/T-	ATG/TAG	ATG/TAA	ATG/TAA	ATG/TAA	ATG/TAA	ATG/TAA
ND1	ATA/TAA	GTG/TAA	GTG/TAA	GTG/T-	ATT/TAA	ATG/TAG	ATG/TAG	ATA/TAA	GTG/TAG	ATA/TAA	ATA/TAA	ATA/TAA	ATA/TAA

Unconventional start codons were highlighted by grey color.

Table S7. Primer pairs used in this study.

No. Fragment	Primer ID	Nucleotide Sequence (5′–3′)	Reference
1	TM-J210	AATTAAGCTATTAGGTTCATACCC	[49]
	TW-N1284	TTAACTTTGAAGGTTAATAGTTT	[49]
2	N2-J586	CCATTCCATTTYTGATTTCC	[49]
	C1-N1738	TTTATTCGTGGAAATGCTATGTC	[49]
3	F-1738	TTCAGCCACCCTATCCCTA	Present study
	R-2130	CGAGTGTCTACATCCATACCA	Present study
4	C1-J2183	CAACATTTATTTTGATTTTTTGG	[49]
	TL2-N3014	TCCAATGCACTAATCTGCCATATT	[49]
5	C1-J2756	ACATTTTTTCCTCAACATTT	[49]
	C2-N3665	CCACAAATTTCTGAACACTG	[49]
6	F-3665	TCTAACCCAAAGTCAGCCC	Present study
	R-4552	GGTCAAATGCTATGATGCC	Present study
7	F-4552	TGAAAGGACTAAGTTACCG	Present study
	R-4792	TATGTGGTTGATGGAGGA	Present study
8	F-4792	ACAAGCCCTCTTTATCAC	Present study
	R-6172	AGGTTATGGATTCTGGGT	Present study
9	TN-J6172	AGAGGCAATTTATTGTTAATAA	[49]
	N5-N7211	TTAAGGCTTTATTATTTATATGTGC	[49]
10	F-7211	GGAAGACCACACAAGGAAAT	Present study
	R-7572	TTATTGGGAGGGACTCTGC	Present study
11	N5-J7572	AAACGGAAACTGAGCTCTCTTAGT	[49]
	N4-N8727	AAATCTTTAATTGCCTATTCTTC	[49]
12	N4-J8641	CCAGAAGAACACAAACCATG	[49]
	N4L-J9629	GTTTGTGAGGGTGCAATAGG	[49]
13	F-9629	CGCACTCAAACGCTCAGGCT	Present study
	R-11335	CTACTGTGAGAAGTTGCCCTAC	Present study
14	CB-J11335	CATATTCAACCAGAATGATA	[49]
	N1-N12067	AATCGTTCTCCATTTGATTTTGC	[49]
15	N1-J11876	CGAGGTAAAGTACCACGTACTCA	[49]
	N1-N12595	GTTGGATTTCTAACTTTATTRGARCG	[49]
16	N1-J12261	TACCTCATAAGAAATAGTTTGAGC	[49]
	LR-N13000	TTACCTTAGGGATAACAGCGTAA	[49]
17	LR-J12888	CCGGTCTGAACTCAGATCATGTA	[49]
	LR-N13889	ATTTATTGTACCTTTTGTATCAG	[49]
18	LR-J13342	CCTTTGCACAGTCAAAATACTGC	[49]
	SR-N14220	TTATGCACACATCGCCCGTC	[49]
19	SR-J14197	GTAAAYCTACTTTGTTACGACTT	[49]
	SR-N14745	GTGCCAGCAAYCGCGGTTATAC	[49]
20	F-14745	ATTAATGAGAGTGACGGGCGAT	Present study
	R-15114	TATGTGGTTGATGGAGGAGTAC	Present study
21	F-15114	ATGTCCTACTATCGTCAC	Present study
	R-210	GGGATGAATGCAATTAGG	Present study

Table S8. Taxa used in this study.

Order	Infroorder	Superfamily	Family	Species	GenBank Accession
Outgroup					
		Pentatomoidea	Cydnidae	*Macroscytus subaeneus*	NC_012457
			Pentatomidae	*Nezara viridula*	NC_011755
Ingroup					
Hemiptera	Cimicomorpha	Reduvioidea	Reduviidae	*Agriosphodrus dohrni*	NC_015842
				Oncocephalus breviscutum	NC_022816
				Sirthenea flayipes	HQ645959
				Triatoma dimidiata	NC_002609
				Valentia hoffmanni	NC_012823
				Brontostoma colossus	NC_024745
				Peirates lepturoides	NC_026672
				Peirates arcuatus	NC_024264
				Reduvius tenebrosus	Present study
		Miroidea	Miridae	*Adelphocoris fasciaticollis*	NC_023796
				Apolygus lucorum	NC_023083
				Adelphocoris nigritylus	NC_027144
				Lygus hesperus	NC_024641
				Lygus lineolaris	NC_021975
				Nesidiocoris tenuis	NC_022677
			Tingidae	*Corythucha ciliata*	NC_022922
				Pseudacysta perseae	KM278221
		Naboidea	Nabidae	*Alloeorhynchus bakeri*	HM235722
				Gorpis annulatus	JF907591
				Gorpis humeralis	NC_019593
				Himacerus apterus	JF927831
				Nabis apicalis	JF907590
		Cimicoidea	Anthocoridae	*Orius niger*	EU427341
